# Obesity-Status-Linked Affecting Factors of Dyslipidemia in Korean Young-Adult Men: Based on the Korea National Health and Nutrition Examination Survey (2019–2021)

**DOI:** 10.3390/healthcare11142015

**Published:** 2023-07-13

**Authors:** Min Kwon, Jinheum Kim, Eunjeong Cha

**Affiliations:** 1 Department of Nursing Science, University of Suwon, Hwaseong-si 18323, Republic of Korea; mink@suwon.ac.kr; 2 Department of Applied Statistics, University of Suwon, Hwaseong-si 18323, Republic of Korea; jinhkim@suwon.ac.kr

**Keywords:** dyslipidemia, young-adult men, waist circumference, body mass index, stress

## Abstract

In recent years, there has been a growing trend of obesity and dyslipidemia among young adult men in South Korea. Therefore, we aimed to identify the obesity-related factors of dyslipidemia among young adult men in Korea using 3-year data (2019–2021) from the 8th Korea National Health and Nutrition Examination Survey. We included 1559 eligible men aged 19–39 years and examined the association between dyslipidemia and participants’ general characteristics, health-related characteristics, and food intake according to obesity status. Statistical analysis was performed using complex sample analysis with weighted household and individual data. The affecting factors of dyslipidemia included age, body image perception, stress, and waist circumference (WC) in the body mass index (BMI) < 25 kg/m^2^ group and age and WC in the BMI ≥ 25 kg/m^2^ group. To prevent and manage dyslipidemia in young adult men, interventions should be developed separately for the normal, underweight, and obese groups. Effective intervention requires measuring WC and focusing on body fat control. Moreover, regular screening of this population should be performed to ensure early diagnosis and management of dyslipidemia.

## 1. Introduction

Dyslipidemia is characterized by abnormal serum lipid concentrations, including those of total cholesterol (TC), triglycerides (TG), low-density lipoprotein cholesterol (LDL-C), high-density lipoprotein cholesterol (HDL-C), or their combinations [1]. The increasing worldwide prevalence of dyslipidemia in the past 30 years has been associated with an increased risk of cardiovascular disease (CVD). Furthermore, dyslipidemia is frequently caused by secondary factors arising from obesity and unhealthy lifestyle habits rather than by primary genetic factors [2]. The World Obesity Federation defines obesity as a chronic, relapsing, and progressive disease process that is caused by excess food, low physical activity, and several other environmental factors that interact with the host’s genetic susceptibility to disrupt energy balance. Body mass index (BMI) and waist circumference can be measured to predict the risk of obesity [3]. The National Institute of Health (NIH) recommends the use of the BMI to define a person as underweight, normal weight, overweight, or obese. Furthermore, the World Health Organization (WHO) classifies obesity into classes I, II, and III (BMI: 30.0–34.9, 35.0–39.9, and ≥40.0, respectively) [4,5]. These BMI classifications have been used by the NIH and WHO for white, Hispanic, and black individuals. However, the cutoffs underestimate the obesity risk in the Asian and South Asian populations, and thus their classification has been slightly altered [6].

Therefore, weight management and healthy lifestyle modifications are essential for the prevention and appropriate management of dyslipidemia, which can significantly alter CVD incidence and mortality rates [7]. Given the absence of specific symptoms, dyslipidemia is frequently neglected. Nevertheless, dyslipidemia has been identified as a major cause of the global increase in CVD prevalence and mortality [8]. Therefore, dyslipidemia should be detected early and managed appropriately [9]. Without therapeutic management, persistent dyslipidemia may induce atherosclerosis and ultimately cause sequelae that could decrease the patient’s quality of life. This highlights the importance of identifying risk factors for dyslipidemia in young adults [10].

The Korea National Health and Nutrition Examination Survey (KNHANES) [11] revealed that the prevalence of hypercholesterolemia in adult men has increased more rapidly than that of other important chronic diseases, including hypertension and diabetes mellitus (DM). The prevalence of hypertension has decreased slightly from 26.6% in 2012 to 25.2% in 2021, and that of DM has increased slightly from 9.4% in 2012 to 12.8% in 2021. In contrast, the prevalence of hypercholesterolemia has increased more than two-fold, from 10.3% in 2012 to 21.5% in 2021. Age- and sex-stratified analyses showed that the prevalence of hypercholesterolemia increased from 15.8% to 22.9% in young adult men and from 11.7% to 14.3% in young adult women during a 3-year period since 2018. This indicates that the prevalence of hypercholesterolemia is increasing at a more alarming pace in young adult men than in young adult women.

KNHANES [11] has also shown that hypercholesterolemia awareness and treatment rates are low at 63.4% and 56.1%, respectively; nonetheless, 86.2% of cases are well managed with pharmacotherapy. Thus, dyslipidemia can be adequately controlled through lifestyle modification and pharmacotherapy following an early diagnosis, which indicates the significance of an accurate diagnosis and proper management. Most importantly, as LDL-C and TG levels continually increase despite the absence of severe hypercholesterolemia in young adults, prolonged exposure could intensify the effects of atherosclerotic CVD [10]. Even in the absence of any risk factors, the National Cholesterol Education Program Adult Treatment Panel III recommends testing for dyslipidemia every 5 years from age 20 onward [12], while its previous guidelines recommended dyslipidemia screening for men and women aged ≥ 35 and 45 years, respectively [11]. With increased life expectancy, cardiovascular health is a vital determinant of an individual’s quality of life, and the identification of risk factors for dyslipidemia in young adulthood could facilitate early management to decrease the lifetime risk of atherosclerotic CVD. The known risk factors for dyslipidemia include old age, male sex, smoking, drinking, stress [13], socioeconomic factors [14], physical activity, diet, and chronic diseases, including hypertension and DM [15]. Several studies have consistently indicated that overweight, obesity, and abdominal obesity constitute the main causes of dyslipidemia [16]. They are the main causes of multiple cardiovascular pathological disorders related to hemodynamic, structural, and functional changes [17]. Moreover, Obesity-related alterations increase the onset of multiple adverse cardiometabolic complications via direct and indirect mechanisms, causing further complications and raising the severity of CVD; thus, various interventions to prevent these problems can help reduce the CVD risk [18]. More recent data highlight abdominal obesity, as determined by waist circumference, as a cardiovascular disease risk marker that is independent of body mass index [19]. However, dyslipidemia also occurs in normal-weight and underweight individuals—groups that may be overlooked owing to the focus on obesity in relation to dyslipidemia. Recent studies have emphasized the increasing prevalence of hypercholesterolemia in underweight individuals [20,21], which necessitates additional research to clarify the above-described association.

We aimed to identify the obesity-related affecting factors of dyslipidemia in young adult men in Korea using the 8th KNHANES (2019–2021) data and provide evidence that could aid the development of preventive interventions and management strategies for dyslipidemia among young Korean men. The primary objective was to examine the association of dyslipidemia with the participants’ general and health-related characteristics, and food intake according to obesity status. The secondary objective was to identify the risk factors of dyslipidemia according to obesity status.

## 2. Materials and Methods

### 2.1. Data and Participants

We conducted a secondary analysis of 3-year data from the 8th KNHANES (2019–2021) [11], a cross-sectional survey conducted by the Korea Disease Control and Prevention Agency in the Korean target population that involved household member verification, health questionnaires, medical examinations, and nutritional assessments. It employed a two-stage stratified cluster sampling method, with the enumeration districts and households as the primary and secondary sampling units, respectively. Data were collected using a rolling sample design, whereby independent samples were extracted for each of the 3 years. Homogeneity of the three rolling samples was ensured through stratification and clustering [22]. The 8th KNHANES enrolled a total of 22,559 participants over 3 years, with 8110, 7359, and 7090 participants in years 1 (2019), 2 (2020), and 3 (2021), respectively. Of the 4960 men aged 19–39 years enrolled during the 3-year survey period, 1559 were diagnosed with dyslipidemia through a blood test performed as part of the KNHANES and had no missing data. After excluding those who had been previously diagnosed and treated, as they might already be undergoing interventions, such as lifestyle modifications, we included 1559 participants in the present study.

### 2.2. Definitions of Variables

#### 2.2.1. Dyslipidemia

According to the Korean Society of Lipid and Atherosclerosis criteria [23], dyslipidemia was defined as the presence of one or more of the following criteria: (1) TC level ≥ 240 mg/dL; (2) TG level ≥ 200 mg/dL; (3) LDL-C level ≥ 160 mg/dL; and (4) HDL-C level < 40 mg/dL. The LDL-C concentration was measured directly; if the TG level was <400 mg/dL, the value was also calculated using Friedewald’s formula.

#### 2.2.2. General Characteristics

Participants’ general characteristics were recorded, including age, education, and income level, which was based on the monthly household income quartiles presented in the KNHANES [11]. The education level was classified as high school or lower and college or higher, as only a small number of participants had middle school or lower education.

#### 2.2.3. Health-Related Characteristics

The assessed health-related characteristics included obesity, waist circumference (WC), body image perception, stress, perceived health, drinking, smoking, walking, muscle training, aerobic physical activity, sleep, work hours, and energy, fat, carbohydrate, and sodium intake. The characteristics were then categorized based on the data collected from the health questionnaire, medical examination, and nutrition survey of the KNHANES [11].

The participants were classified as obese (BMI ≥ 25 kg/m^2^), nonobese (BMI < 25 kg/m^2^), and those with a WC of ≥90 and <90 cm [24]. Body image perception was stratified as that of being skinny, normal, or overweight. Participants who reported little or no stress in daily life were considered to have “no stress”, whereas those who reported moderate to high stress levels were considered to have “stress.” Perceived health was classified as good, fair, or poor. Based on the average daily alcohol consumption, the participants were classified as nondrinkers, moderate drinkers, and heavy drinkers [25]. The average daily alcohol intake was calculated by multiplying the yearly frequency of alcohol consumption by the amount consumed in a single drinking episode and further dividing the obtained value by 30. To facilitate calculations, the alcohol consumption frequency categories of “never”, “less than once a month”, “about once a month”, “2–4 times a month”, “2–3 times a week”, and “≥4 times a week” were converted to “0 times a month”, “0.5 times a month”, “once a month”, “3 times a month”, “10 times a month”, and “16 times a month”, respectively. Additionally, the amount consumed in a single drinking episode was categorized as “1–2 drinks”, “3–4 drinks”, “5–6 drinks”, “7–9 drinks”, and “10 or more drinks”; however, for the analysis in this study, the amounts were converted to 1.5, 3.5, 5.5, 8.0, and 10.0 standard drinks, respectively. The WHO [26] defines one standard drink as containing 10 g of pure alcohol; therefore, the number of drinking occasions and the amount of alcohol in a standard drink (10 g) were multiplied and divided by 30 to calculate the average daily alcohol intake [27]. Participants who had never smoked were categorized as nonsmokers, and former/current smokers were categorized as smokers. Physical activity [28] was recorded as “yes” if the individual had walked at least 10 min a session, 30 min a day, and 5 days a week in the past week. Aerobic physical activity was recorded as “yes” if the individual had engaged in at least 2.5 h of moderate-intensity activity or 1.25 h of vigorous-intensity activity per week or an equivalent combination of both (1 min of vigorous-intensity activity = 2 min of moderate-intensity activity). Strength training was recorded as “yes” if the participants had engaged in strength training exercises on at least 2 days in the past week. Sleep duration was classified as <7 or ≥7 h [29], and work hours were determined based on the average weekly working hours.

The participants’ dietary intake was assessed using a 24-h dietary recall survey and, based on the 2020 Korean Dietary Reference Intakes [30], was classified as follows: total energy intake (<3125 kcal, adequate; ≥3125 kcal, excessive); sodium intake (≤2000 mg, adequate; >2000 mg, excessive). The acceptable macronutrient distribution range (AMDR) for carbohydrates and fat from the total energy intake was calculated. Carbohydrate intake was classified as adequate or excessive if the AMDR was ≤65% and >65%, respectively, and fat intake was classified as adequate or excessive if the AMDR was ≤ 30% and >30%, respectively.

### 2.3. Ethical Considerations

KNHANES data are openly available, and the participant data were anonymized prior to their publication. This study was exempted from review (USW IRB/2303-045-02) by the review board of the institution the authors are affiliated with.

### 2.4. Statistical Analysis

Statistical analyses were performed using SAS version 9.4 and R version 4.2.3, and household- and individual-weighted values were used for complex sample analysis. Participants’ general characteristics and health-related characteristics were analyzed with complex sample frequency analysis and descriptive statistics (mean and standard error).

Differences in dyslipidemia according to the general and health-related characteristics of the obese and nonobese groups were analyzed with the complex-sample chi-square test. Using a complex-sample multivariate logistic regression analysis that included all risk factors, the factors that affected dyslipidemia in the obese and nonobese groups were analyzed separately. *p* < 0.05 was deemed statistically significant. The validity of the predictive model was evaluated by generating calibration plots for each obesity group using the Hosmer–Lemeshow method [31], and the area under the curve (AUC) was calculated and compared using the roc.test function in the pROC R package [32].

## 3. Results

### 3.1. Dyslipidemia Prevalence by General and Health-Related Characteristics in Obese and Nonobese Groups

Of the 1559 participants, 861 (55.2%) had a BMI < 25 kg/m^2^, and 698 (44.8%) had a BMI ≥ 25 kg/m^2^. The prevalence of dyslipidemia was 20.7% and 44.6% in the BMI < 25 and BMI ≥ 25 kg/m^2^ groups, respectively. Dyslipidemia prevalence differed significantly according to age, body image perception, stress, aerobic exercise, and WC in the BMI < 25 kg/m^2^ group and according to age, walking, muscle training, sleep, and WC in the BMI ≥ 25 kg/m^2^ group (Table 1).

### 3.2. Affecting Factors of Dyslipidemia According to Obesity Status

We used multivariate logistic regression to separately analyze factors that influenced dyslipidemia in both groups (Table 2). The significant factors for dyslipidemia included age, body image perception, stress, and WC in the BMI < 25 kg/m^2^ group and age and WC in the BMI ≥ 25 kg/m^2^ group. In both groups, the risk of dyslipidemia increased with increasing age. In the BMI < 25 kg/m^2^ group, the risk for dyslipidemia was 2.67 times higher among those who perceived themselves to be overweight than among those who perceived themselves to be skinny (odds ratio [OR], 2.67; 95% confidence interval [CI], 1.39–5.11; *p* = 0.009).

The risk of dyslipidemia was higher in participants with stress (OR, 1.65; 95% CI, 1.05–2.62; *p* = 0.032) and in those with a WC ≥ 90 cm (OR, 7.48; 95% CI, 2.72–20.59; *p* < 0.001). In the BMI ≥ 25 kg/m^2^ group, the risk for dyslipidemia was higher in the WC ≥ 90 cm group than in the WC < 90 cm group (OR, 1.67; 95% CI, 1.06–2.62; *p* < 0.028).

### 3.3. Model Fit

**Figure 1:**
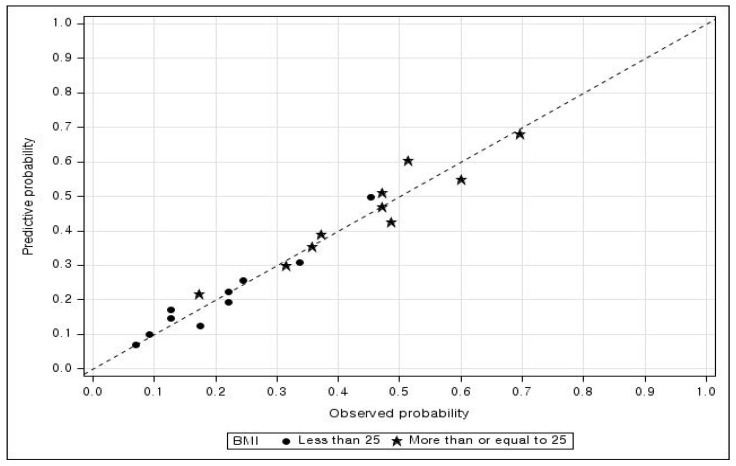
Calibration plots by BMI < 25 kg/m^2^ and ≥25 kg/m^2^. BMI, body mass index.

**Figure 2:**
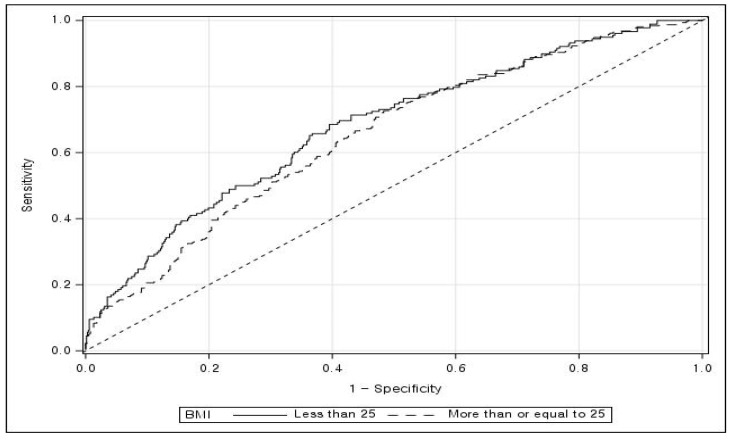
Receiver operating characteristic curves by BMI < 25 kg/m^2^ and ≥25 kg/m^2^. BMI, body mass index.

The coefficient of determination (Nagelkerke’s R^2^) [33] for the BMI < 25 kg/m^2^ and ≥25 kg/m^2^ groups was 13.7% and 10.1%, respectively. The concordant pair rates were 67.8% and 65.1%, respectively, and accuracy at the same cutoff value of 0.5 was 80.3% and 60.9%, respectively. Figure 1 presents a calibration plot that lists the predicted probabilities in descending order and is divided into 10 equal groups. With regard to the outliers that deviate substantially from the reference line (dashed line), there were more outliers in the BMI ≥ 25 kg/m^2^ group (star-shaped) than in the BMI < 25 kg/m^2^ group (circle-shaped). Figure 2 presents the receiver operating characteristic curve, which was plotted by varying the threshold from 0 to 1. The curve for the BMI < 25 kg/m^2^ group (solid line) is further away from the reference line (dashed line) than the curve for the BMI ≥ 25 kg/m^2^ group (dotted line). The AUC values were 0.68 and 0.65 for the BMI < 25 and ≥25 kg/m^2^ groups, respectively. To compare the AUCs (or ROC curves) of the obese and non-obese groups, the value obtained with Delong et al.’s D test [34] was 0.90 (*p* = 0.368), and the value of 2000 bootstraps was 0.93 (*p* = 0.352). Thus, no statistically significant intergroup difference in the AUC (or ROC curves) was detected.

## 4. Discussion

This study was conducted to identify the affecting factors of dyslipidemia in obese and nonobese young adult men in South Korea using the data from the 8th KNHANES (2019–2021) [22] and to provide evidence that could facilitate the development of intervention strategies for the prevention and management of dyslipidemia in this population. The prevalence of dyslipidemia in this study was 20.7% in the BMI < 25 kg/m^2^ group and 44.6% in the BMI ≥ 25 kg/m^2^ group. A previous study [35] revealed a dyslipidemia prevalence of 45.6% in the total adult male population, with rates of 26.6% and 40.8% in the 20–29 and 30–39 age groups, respectively, similar to the prevalence of 31.5% in the whole study cohort, with varying prevalence across age groups. These results highlight the need for dyslipidemia management in young adult men. Domanski et al. [36] and Shapiro et al. [37] reported that even if the LDL-C concentration is decreased in middle adulthood or later, the long-term risk for CVD is markedly influenced by elevated LDL-C levels in young adulthood. Thus, the risks accumulated during the early stages of exposure remain largely uneliminated, indicating the significance of early intervention for lowering LDL-C levels.

In our study, the affecting factors of dyslipidemia in the BMI < 25 kg/m^2^ group were age, body image perception, stress, and WC. Other studies [13,15] have reported that the prevalence of dyslipidemia increases with increasing age, which is consistent with our findings. This association can be explained by an increased rate of comorbidities, such as obesity, hypertension, and DM, with increasing age. Compared to those who perceived themselves to be skinny, those who perceived themselves as being normal or overweight had 1.90 and 2.67 times higher odds for dyslipidemia, respectively. Body image perception is a psychosocial factor that may influence body weight [38] and reportedly has a greater impact than BMI on weight control-related behaviors [39]. This may be associated with individuals’ awareness of their body fat percentage regardless of whether they have normal or underweight BMI, which is supported by reports that Asians have a higher perceived body fat percentage in relation to their actual BMI as compared with that in Western populations [20,40]. In line with these findings, our study revealed that among normal or underweight individuals, a WC ≥ 90 cm was associated with a 7.48-fold increase in the odds of dyslipidemia as compared with a WC < 90 cm. WC was also identified as an affecting factor in dyslipidemia by Choi et al. [41] in men aged 19–59 years and by Chong [42] in young adults. However, Chong [42] reported a lower OR of 2.57, which may be attributed to their combined analysis of young adult men and women. In our study, there was a considerable asymmetric distribution of participants in the WC < 90 cm (n = 834) and WC ≥ 90 cm (n = 27) groups, which may have contributed to the higher OR; thus, this finding necessitates further research. WC is a common measure of abdominal fat accumulation and is strongly associated with cardiovascular mortality [43]. Abdominal obesity, in particular, induces insulin resistance and increases the risk of metabolic syndrome [44,45]. Therefore, in addition to maintaining a healthy body weight, young adult men require intensive management for a healthy lifestyle to maintain a healthy WC.

Stress was identified as a predictor of dyslipidemia in the normal and underweight groups. This finding is consistent with that of Tenk et al.’s work [46], who observed that mental health and stress were associated with dyslipidemia, but inconsistent with that of Choi et al.’s work [41] for adult men and Chong [42] for young adults. This discrepancy may be attributed to the specific focus on young adult men based on BMI in our study. Therefore, stress management may be essential for young adult men with a normal or underweight BMI to reduce the prevalence of dyslipidemia.

In the group with a BMI ≥ 25 kg/m^2^, both age and WC were identified as affecting factors of dyslipidemia, which is consistent with the findings of many previous studies [13,15,41,42]. Other healthy lifestyle-related factors had no significant influence on dyslipidemia. Considering the findings from the WHO MONICA Project, which identified obesity as the strongest risk factor for hypercholesterolemia in young adults [47], controlling body weight and fat may be the most effective strategy for reducing dyslipidemia in young adult men with a BMI ≥ 25 kg/m^2^.

Chong [42] reported that dyslipidemia could be predicted by the perceived health status, BMI, and dining-out frequency in the entire adult population; by the perceived health status and WC in young adulthood (19–44 years); and by the perceived health status in middle adulthood (45–64 years) and older adulthood (≥65 years). Choi et al. [41] showed that marital status, obesity, abdominal obesity, smoking, physical activity, hypertension, DM, and perceived health status are factors affecting dyslipidemia in adult men aged 19–59 years. Further, Jeon et al. [15] identified age, education level, drinking, smoking, physical activity, hypertension, and DM as affecting factors of dyslipidemia among adults aged ≥ 20 years and older men (≥65 years). However, previously identified lifestyle risk factors for dyslipidemia, namely smoking, drinking, physical activity, and food intake [13,15,41], were not significant factors in our study; therefore, further research is necessary.

Based on the findings of previous studies and the present study, effective management of dyslipidemia requires the identification of relevant factors based on the sociodemographic characteristics of the target population and health interventions based on these factors. However, in reality, the management of dyslipidemia is primarily targeted toward middle-aged and obese populations. Nevertheless, our study reaffirmed the importance of managing dyslipidemia in young adult men and highlighted the need for dyslipidemia management in the normal and underweight BMI groups as well. One key significance of this study is that we identified affecting factors specific to each target group, thus providing insight into the development of interventions for dyslipidemia in young adult men. Finally, to enhance the reliability of the statistical estimations, we merged 3 years of annual data from 2019 to 2021 and converted the year-based weights into combined weights for the analysis.

The limitations of this study are as follows: First, although the KNHANES provides a reliable large-scale dataset representative of the Korean population, the cross-sectional survey design prevents the assessment of the long-term effects of factors, such as lifestyle, on the incidence of dyslipidemia in a young adult population. Additionally, causality cannot be determined using these data. Second, the nutrition survey relies on a 24-h dietary recall, which requires the participant to recall the types and amounts of foods consumed in the past 24 h; thus, these data cannot provide objective information on an individual’s usual nutrient intake. Third, our study involved secondary data analysis; therefore, we could not identify other lifestyle-related factors in young adult men. To overcome the limitations of this study, we recommend further replication and longitudinal studies that include lifestyle and health behavior factors and assess nutrient intake using a more accurate nutrition survey. Furthermore, we recommend additional studies for the WHO-based classification of obesity (classes I, II, and III) and further investigation, through diverse study designs and analysis methods, of the underlying factors for obesity in each class while evaluating obesity—a potent risk factor for dyslipidemia—as the mediating variable. We evaluated TC, TG, HDL-C, and LDL-C based on the serum lipid concentration to identify the factors associated with dyslipidemia in our population of interest. However, future studies should also include lipoprotein (a), which was recently identified as an independent causal risk factor for CVD [48,49]. To compare whether risk factors have the same effect on dyslipidemia depending on obesity status, instead of our proposed separate models, we recommend research using a combined model that includes the interaction effects between obesity status and risk factors. In cases wherein there is an imbalance in the population (e.g., non-obese group: 79.3% vs. 20.7%), accuracy, as a measure of the predictive model, might generate non-informative results. Therefore, precision, recall (or sensitivity), or F1 [50] can be utilized instead. Moreover, by using the resampling method proposed by Menardi and Torelli (2014) [51], accuracy can be recalculated after ensuring that the two groups for the binary variable (dyslipidemia or no dyslipidemia) are similar.

## 5. Conclusions

The prevention and management of dyslipidemia in young adult is imperative for CVD prevention and management, and necessitates interventional strategies that are customized for the young adult male population that has different BMI classifications. In the normal and underweight BMI groups, WC may be a more suitable measure of abdominal obesity and regulating body fat than BMI. Furthermore, interventions for fostering a more positive body image, increasing motivation for body fat management, and managing stress are crucial for these population groups. For patients with obesity, interventions that prioritize weight and body fat control are needed. Furthermore, educational strategies to increase the individual awareness of the importance of early detection and management of dyslipidemia should be undertaken and regular screening should be promoted.

## Figures and Tables

**Table 1:** Summary statistics of dyslipidemia and significance of dyslipidemia in subgroups stratified by body mass index (BMI) < 25 and ≥25 kg/m^2^.

Variable	Category	BMI, kg/m^2^					
		<25			≥25		
		Dyslipidemia		Rao–Scott Chi-Squared Test (*p*-Value)	Dyslipidemia		Rao–Scott Chi-Squared Test (*p*-Value)
		No	Yes		**No**	**Yes**	
		n (% ^1^)	n (% ^1^)		**n (% ^1^)**	**n (% ^1^)**	
Total		683 (79.3)	178 (20.7)	-	387(55.4)	311 (44.6)	-
Age, years		28.5 ± 0.27 ^2^	30.5 ± 0.47 ^2^	−4.22 (<0.001) ^3^	29.5. ± 0.35 ^2^	31.7 ± 0.37 ^2^	−4.49 ( < 0.001) ^3^
Education	High school or below	354 (81.9)	74 (18.1)	2.83 (0.0924)	182 (59.1)	121 (40.9)	2.18 (0.1402)
	College or above	329 (76.6)	104 (23.4)		205 (52.8)	190 (47.2)	
Income	1st quartile	155 (75.3)	47 (24.7)	1.89 (0.5963)	98 (52.9)	88 (47.1)	1.75 (0.6250)
	2nd quartile	179 (79.9)	48 (20.1)		99 (53.5)	86 (46.5)	
	3rd quartile	166 (80.6)	42 (19.4)		101 (60.2)	69 (39.8)	
	4th quartile	183 (80.6)	41 (19.4)		89 (55.9)	68 (44.1)	
Body image perception	Skinny	234 (87.1)	32 (12.9)	15.88 (0.0004)	-	-	0.18 (0.6678)
	Normal	333 (78.1)	95 (21.9)		46 (58.1)	31 (41.9)	
	Overweight	116 (69.1)	51 (30.9)		341 (55.2)	280 (44.8)	
Stress	No	510 (81.7)	119 (18.3)	7.13 (0.0076)	254 (57.8)	183 (42.2)	1.87 (0.1719)
	Yes	173 (72.4)	59 (27.6)		133 (51.7)	128 (48.3)	
Subjective health	Good	332 (79.7)	86 (20.3)	0.40 (0.8196)	148 (57.8)	104 (42.2)	4.96 (0.0836)
	Normal	297 (79.3)	79 (20.7)		191 (56.9)	153 (43.1)	
	Bad	54 (75.9)	13 (24.1)		48 (44.9)	54 (55.1)	
Drinking	No	64 (81.4)	18 (18.6)	0.93 (0.6267)	30 (46.6)	36 (53.4)	1.91 (0.3844)
	Moderate	550 (78.5)	145 (21.5)		297 (56.4)	229 (43.6)	
	High	69 (82.7)	15 (17.3)		60 (56.7)	46 (43.3)	
Smoking	No	294 (81.8)	65 (18.2)	1.83 (0.1765)	152 (58.9)	111 (41.1)	1.35 (0.2446)
	Yes	389 (77.4)	113 (22.6)		235 (53.5)	200 (46.5)	
Walking	No	361 (76.6)	105 (23.4)	3.28 (0.0702)	198 (49.9)	189 (50.1)	8.84 (0.0029)
	Yes	322 (82.3)	73 (17.7)		189 (61.9)	122 (38.1)	
Muscle exercise	No	382 (77.1)	112 (22.9)	2.53 (0.1116)	248 (52.3)	224 (47.7)	4.08 (0.0435)
	Yes	301 (82.0)	66 (18.0)		139 (61.5)	87 (38.5)	
Aerobic exercise	No	266 (75.5)	82 (24.5)	3.95 (0.0468)	143 (50.9)	143 (549.1)	3.04 (0.0814)
	Yes	417 (81.7)	96 (18.3)		244 (58.5)	168 (41.5)	
Sleep	Insufficient	454 (80.0)	117 (20.0)	0.39 (0.5308)	237 (57.1)	187 (42.9)	0.72 (0.3951)
	Normal	229 (77.7)	61 (22.3)		150 (53.2)	124 (46.8)	
Working hours, minutes		33.7 ± 0.87 ^2^	35.6 ± 1.72 ^2^	−1.06 (0.2909) ^3^	35.4 ± 1.19 ^2^	38.1 ± 1.25 ^2^	−1.54 (0.1236)^3^
Waist circumference, cm	<90	674 (80.9)	160 (19.1)	33.71 (<0.001)	131 (66.9)	60 (33.1)	10.11 (0.0015)
	≥90	9 (33.0)	18 (67.0)		256 (51.5)	251 (48.5)	
Energy intake	Normal	544 (78.7)	147 (21.3)	0.42 (0.5176)	313 (56.4)	250 (43.6)	0.90 (0.3421)
	Excessive	139 (81.3)	31 (18.7)		74 (51.6)	61 (48.4)	
Fat intake	Normal	456 (78.8)	127 (21.2)	0.19 (0.6647)	278 (55.4)	226 (44.5)	0.01 (0.9375)
	Excessive	227 (80.2)	51 (19.8)		109 (55.8)	85 (44.2)	
Carbohydrate intake	Normal	557 (78.7)	152 (21.2)	0.44 (0.5092)	324 (56.6)	257 (43.4)	1.47 (0.2260)
	Excessive	126 (81.5)	26 (18.5)		63 (49.6)	54 (50.4)	
Sodium intake	Normal	93 (79.1)	27 (20.9)	0.0003 (0.9855)	60 (63.4)	36 (36.6)	2.25 (0.1338)
	Excessive	590 (79.2)	151 (20.8)		327 (54.2)	275 (45.8)	

^1^ The values were calculated using pooled weights. ^2^ mean ± standard error. ^3^ *t*-test.

**Table 2:** Results of multivariate logistic regression on dyslipidemia of participants by body mass index (BMI) <25 and ≥25 kg/m^2.^.

Variable	Category	BMI, kg/m^2^			
		<25		≥25	
		OR (95% CI)	*p*-Value	OR (95% CI)	*p*-Value
Age, years		1.04 (1.00–1.09)	0.0434	1.06 (1.02–1.11)	0.0030
Education (Ref: High school or below)	College or above	1.05 (0.68–1.64)	0.8187	1.01 (0.65,1.60)	0.9692
Income (Ref: 1st quartile)	2nd quartile	0.65 (0.36–1.18)	0.4318	0.97 (0.59–1.60)	0.4869
	3rd quartile	0.71 (0.40–1.28)		0.69 (0.41–1.17)	
	4th quartile	0.67 (0.39–1.16)		0.97 (0.58–1.63)	
Body image perception (Ref: Skinny)	Normal	1.89 (1.12–3.18)	0.0093	-	0.7979
	Overweight	2.66 (1.40–5.08)		0.92 (0.49–1.73) **^1^**	
Stress (Ref: No)	Yes	1.64 (1.05–2.59)	0.0329	1.05 (0.71–1.54)	0.8226
Subjective health (Ref: Good)	Normal	0.76 (0.50–1.18)	0.3419	0.80 (0.54–1.18)	0.1721
	Bad	1.22 (0.55–2.71)		1.21 (0.68–2.14)	
Drinking (Ref: No)	Moderate	1.13 (0.57–2.23)	0.4187	0.66 (0.35–1.23)	0.2442
	Heavy	0.71 (0.29–1.77)		0.52 (0.24–1.12)	
Smoking (Ref: No)	Yes	1.17 (0.77–1.79)	0.4668	1.21 (0.77–1.90)	0.4082
Walking (Ref: No)	Yes	0.78 (0.51–1.18)	0.2332	0.72 (0.50–1.05)	0.0886
Muscle exercise (Ref: No)	Yes	0.89 (0.57–1.37)	0.5864	0.980 (0.65–1.48)	0.9273
Aerobic exercise (Ref: No)	Yes	0.81 (0.53–1.26)	0.3515	0.91 (0.61–1.35)	0.6332
Sleep (Ref: Normal)	Insufficient	1.08 (0.68–1.73)	0.7342	1.09 (0.74–1.60)	0.6571
Working hours, minutes		1.00 (0.99–1.01)	0.7310	1.00 (0.99–1.01)	0.9967
Waist circumference (Ref: <90 cm)	≥90 cm	7.48 (2.70–20.79)	<0.001	1.69 (1.08–2.64)	0.0231
Energy intake (Ref: Normal)	Excessive	0.92 (0.44–1.90)	0.8119	1.04 (0.54–2.02)	0.9005
Fat intake (Ref: Normal)	Excessive	1.20 (0.71–2.03)	0.5049	0.96 (0.56–1.63)	0.8763
Carbohydrate intake (Ref: Normal)	Excessive	0.78 (0.41–1.49)	0.4512	1.16 (0.65–2.06)	0.6140
Sodium intake (Ref: Normal)	Excessive	1.06 (0.61–1.84)	0.8434	1.18 (0.68–2.04)	0.5611
Model fit measures	Nagelkerke R^2^	0.137		0.101	
	Concordant pairs (%)	67.8		65.1	
	AUC (95% CI)	0.680 (0.636–0.724)		0.652 (0.612–0.693)	
	Accuracy	0.803		0.609	

OR, odds ratio; CI, confidence interval. ^1^ Ref: normal.

## Data Availability

The data that support the findings of this study are available from the corresponding author upon reasonable request.
